# Generation of patterns and higher harmonics in 1D quantum droplets in tilted and driven quasi-periodic confinements

**DOI:** 10.1038/s41598-024-73319-6

**Published:** 2024-10-15

**Authors:** Maitri R. Pathak, Jayanta Bera, Utpal Roy, Ajay Nath

**Affiliations:** 1 Indian Institute of Information Technology Vadodara Gujarat India, Gandhinagar, 382 028 India; 2 C. V. Raman Global University, Bhubaneswar, Odisha 752 054 India; 3 Indian Institute of Technology Patna, Patna, Bihar 801 106 India

**Keywords:** Quantum droplets, Bi-periodic optical lattices, Higher harmonics, Atomic and molecular physics, Ultracold gases

## Abstract

The generation of patterns by breaking the spatial symmetry in external confinement is a captivating area of physics. The emergence of patterns is a fundamental inquiry spanning various disciplines such as nonlinear optics, condensed matter physics, and fluid dynamics. The article investigates the generation of a variety of patterns in a one-dimensional binary mixture of Bose–Einstein condensate forming quantum droplets. By solving the extended Gross–Pitaevskii equation in the presence of tilted and driven engineered bi-chromatic optical lattices (BOL), the out-of-equilibrium dynamics of droplets under strong dc and ac fields are illustrated. Under the influence of a dc field, a stripe-like pattern emerges in the temporal domain, while the scenario with ac fields demonstrates temporal periodic and bi-periodic oscillations of density waves. The width and period of formed patterns are directly correlated with the strength of ac and dc fields. Moreover, temporal modulation of the BOL potential depth yields various harmonics in the oscillations of the condensate density pattern. Through Fast Fourier Transform (FFT) analysis, it is confirmed that these harmonics encompass multiple and combinational frequencies, suggesting potential applications in generating desired frequency combs within quantum droplets. We have also carried out a thorough numerical stability check of the obtained solutions and found them sufficiently stable.

## Introduction

Spontaneous pattern formation is a widespread phenomenon in nature^1^. Examples of pattern formation include flocks of birds in flight, soliton trains in shallow water, the disturbance created by dropping pebbles in water, and the formation of sand dune ripples. These macroscopic patterns emerge from driving systems out of equilibrium, leading to the appearance and disappearance of transient states and the formation of various patterns with a homogeneous background. Investigating the emergence of patterns by disrupting the spatial symmetry of external potential landscapes is a fascinating area of physics research^1,2^. The occurrence of order in non-equilibrium scenarios has spurred extensive experimental and theoretical research in various fields such as quantum ferrofluids^3^, superfluid helium^4^, optically nonlinear media^5^, turbulent Rayleigh–Bénard convection^6^, condensed matter physics^7^, hydrodynamics^1^, biological matter^8–11^, and cosmology^12^. These phenomena can be explored in the quantum realm using ultra-cold atoms. In this context, periodically driven Bose–Einstein condensates (BECs) have been a playground for studying various pattern formation phenomena such as bright soliton trains^13^, Faraday waves^14^, time-crystal phases in Floquet-driven systems^15,16^, the generation of super-solid to super-glass phases in quantum ferrofluids^17^, generation of higher harmonics^18^ and the formation of super-solid stripe patterns in dipolar BEC^19,20^. In the BEC phase, various pattern morphologies emerge either due to periodic driving^21^ or as a result of long-range interactions, as observed in the super-solid stripe pattern^22^. Particularly in periodic optical lattice confinement, a variety of density wave morphologies are observed by disrupting the spatial symmetry of the underlying lattice. This is demonstrated in scenarios such as tilted Bose–Hubbard models^23^ or spin chains created in Rydberg-atom arrays^24^.

In recent times, quantum droplets (QDs), a novel liquid phase characterized by densities that can be up to eight orders of magnitude more dilute than liquid helium droplets has been experimentally realized in one component dipolar^20,25^ and Bose–Bose BEC mixture^26^. QDs represent the only atomic species that naturally maintains liquidity at zero temperature and achieve stable self-localization through their inherent nonlinearity^22,27^. These self-binding of droplets is predicted to exist through a delicate balance between the effective mean field (EMF) between atom–atom interactions and the beyond mean field (BMF) effects due to quantum fluctuations^28–30^. The BMF effect is represented by extended Gross–Pitaevskii equation (eGPE) model by incorporating the first order Lee–Huang–Yang (LHY) corrections in it which in the context of the effectively one-dimensional scenario introduces an attractive quadratic nonlinear term^29,31^. Explorations into QDs have garnered considerable attention across diverse contexts within the BEC literature^32–56^. Further, the QDs predicted to exist in 1D Bose–Hubbard mixtures in optical lattices for small and large interaction strengths across a wide range of interaction strengths^57^. The interaction between these nonlinearities and periodic optical lattices, as highlighted in studies by Morera et al.^58^, Pathak et al.^59,60^, Zhou et al.^61^, Nie et al.^62^, Zhao et al.^63^, Dong et al.^64^, Zheng et al.^65^, Zhang et al.^66^ and Zhang et al.^15^ could be of particular interest and uniqueness, as the properties of lattice solitons are heavily influenced by the nature and regulation of nonlinear interactions within the system. Though lots of emphasis on investigating the structural profile and stability of QDs in the recent past, only a limited number of works are available in the literature that focuses on the dynamical aspect of QDs in the non-equilibrium scenario. Especially, the pattern formation on QDs in presence of periodically driven or tilted quasi-periodic OL like bi-periodic OL (BOL) is unexplored. The main challenge in studying this phenomenon stems from the complexity of analytically understanding the precise dynamics of droplets under these conditions. Additionally, observing the diverse patterns and linking them to specific trap parameters will uncover novel insights into the physics of droplet domains.

Motivated from that, the article introduces an analytical framework to study the droplet dynamics under tilted and driven bi-chromatic optical lattice (BOL) confinement in a 1D setting. We consider a weakly interacting binary BEC mixture with components having identical masses and number of atoms in the chosen confinement. The resultant competing repulsive cubic EMF and attractive BMF interactions are studied in 1D eGPE model and exact form of wavefunction calculated. Utilizing the form of the wave function, we investigate the temporal evolution of condensate density for the changing driving frequency (dc and ac fields) and magnitude of potential depths of BOL confinement. We observe that for a small magnitude of potential depth in both dc and ac fields, the system remains in the droplet phase whereas an increase in potential depth results in the fragmentation of the droplet profile leading to the generation of supersolid phase and gap solitons in between the BOL lattices. In the dc field, the temporal perturbation results in the formation of a stripe lattice-like pattern on the droplet in the temporal domain whereas ac temporal periodic and bi-periodic perturbation of potential results in the generation of temporal supersolid-like periodic lattice and superlattice density patterns on QDs. The width and period of formed patterns are directly correlated with the strength of ac and dc fields. Moreover, temporal modulation of the BOL potential depth yields various harmonics in the oscillations of the condensate density pattern. Through FFT analysis, it is confirmed that these harmonics encompass multiple and combinational frequencies, suggesting potential applications in generating desired frequency combs within quantum droplets. We have conducted the numerical stability analysis of the solutions under two specific scenarios, such as dynamical stability and structural stability. It is observed that our analytical solutions are sufficiently stable for both types of noise inclusions.

## Results

### The model and analytical framework

We begin with by considering 1D homonuclear binary BEC mixture under the time modulated external trap of the form:

1 $$\begin{aligned} V(x,t)= V_{1}(t) x + V_{2}\cos [2l\{x - \delta (t)\}] + V_{3}\cos [l\{x-\delta (t)\}] + V_{4} \; \exp [2 \beta (t) \cos [l\{x-\delta (t)\}]], \end{aligned}$$

which is a combination of the linear trap with two optical lattices having commensurate lattice periods and an exponential periodic trap. Here, $\beta (t)$ is a time dependent function and the presence of an exponential term in the above equation makes the form of trap a tilted and driven multi-color optical lattice^59^. Using Taylor expansion with the approximation that $-1<\beta (t)<1$, then tilted and driven BOL confinement obtained from Eq. (1):

2 $$\begin{aligned} V(x,t) \simeq V_{1}x+ V_{2}\cos [2l\{x - \delta (t)\}] + V_{3}^{\prime }\cos [l\{x - \delta (t)\}], \end{aligned}$$

where $\delta (t)$ is the temporal driving field acting on the system and $V_{3}^{\prime }=V_{3}+2 \beta (t) V_{4}$.

Here, we consider the binary BEC in the two distinct hyperfine states of ^39^K^67^ in one-dimensional geometry. Here, the transversal directions are tightly confined, while the x-direction remains elongated and unconfined. For simplicity, both states of binary BEC mixture are mutually symmetric such that: $\psi _{1}=\psi _{2}=c_{0} \psi$ and share same atom number (i.e. $N_{1}$ = $N_{2}$ = *N*) with equal masses. Further, we consider that the intra-atomic coupling constants equal such that: $g_{11} = g_{22}\equiv g (>0)=2 \hbar ^{2} a_{s}(x, t)/(m a^{2}_{\perp })$ whereas inter-atomic coupling constant $g_{12} (<0)$ which provides droplet environment for $\delta g = g_{12} + g > 0$^38^. These assumptions make the participating components identical, allowing the binary BEC mixture to be characterized by the following simplified single-component dimensionless 1D eGPE, which includes the first-order LHY quantum correction^29,38^:

3 $$\begin{aligned} i\frac{ \partial \psi }{\partial t} =- \frac{1}{2} \frac{ \partial ^2 \psi }{\partial x^2} - g_1(x,t) |\psi |\psi + g_2(x,t) |\psi |^2\psi + V(x,t)\psi + i \tau (t) \psi , \end{aligned}$$

where $\psi$ is 1D wavefunction. In this, $g_{1}(x, t)$ and $g_{2}(x, t)$ are non-zero functions representing the BMF and EMF coupling strengths of the binary BEC mixture, and importantly the quadratic nonlinearity reflects the 1D attractive aspect of the LHY contribution, while the cubic term addresses the conventional mean-field repulsion. Here, $\tau (t)$ is the temporally varying gain or loss of the condensate atoms in BEC and can be tuned by: (a) inelastic collisions^68^, dipolar relaxation^69^, (b) interaction with thermal component^70^, and (c) through collapse dynamics^71^. Physically, for $\tau (t)>0$, atoms are pumped into the condensate either from a reservoir or from thermal background whereas $\tau (t)<0$ corresponds to a loss of condensate atoms due to dipolar relaxation^72^.

Also, the wavefunction, length, and time are in units of $c_{0}=(2 \sqrt{g})^{3/2}/(\pi \xi (2|\delta g|)^{3/4}$, $\xi$, and $\hbar ^{2}/m \xi ^{2}$ respectively with $\xi =\pi \hbar ^{2} \sqrt{|g|}/(m g \sqrt{2}g)$ is the healing length of the system^38^. The typical evolution times examined in this study are on the order of $t \approx 10^{3}$, corresponding to approximately 800 ms, for example, with a transverse confinement $\omega _{{\perp }} \approx 200$ Hz as utilized in the experiment cited in reference^67^. In the Eq. (1), $l = 2 \pi a_{\perp }/ \lambda$ is the scaled lattice wave vector, which is commensurate for the two optical lattices (*l*, 2*l*) forming BOL trap with $a_{\perp }=\sqrt{\hbar /m \omega _{\perp }}$, and $\omega _{\perp }$ is the transverse oscillator frequency. Here, $V_{1}(t)$ is the time-dependent amplitude of the linear trap generating tilt in the chosen trap combination, and $V_{j}$ ($j=2,3,4$) represents the potential depths of each OL and is connected with the recoil energy: $E_{R}=\frac{2\pi ^{2}\hbar ^{2}}{m \lambda ^{2}}$. Here, $\lambda$, $\hbar$ and mass *m* represents the laser’s wavelength, reduced Plack’s constant, and mass of binary BEC atoms^59,73^. Experimentally, the BOL confinement is realized by the superposition of two optical lattices on each other^74^ and in Eq. (2) similar condition is chosen.

Equation (3) is the 1D eGPE model which accommodates various nonlinear excitations, including dark solitons, kinks, droplets, bubbles, as well as stationary periodic waves of the Jacobi-elliptic type^29–31,75–78^. In order to obtain analytical solution of Eq. (3) for investigating the spatio-temporal dynamics of QDs in the chosen time modulated tilted and driven BOL confinement, we choose the following ansatz solution based on the similarity transformation method^79^:

4 $$\begin{aligned} \psi (x,t)= \rho (x,t)F[\eta (x,t)]e^{i\phi (x,t)}, \end{aligned}$$

and connect it with the solvable second order differential equation:

5 $$\begin{aligned} -F_{\eta \eta }-G_1\mid F(\eta )\mid F +G_2\mid F(\eta )\mid ^{2} F=\mu F, \end{aligned}$$

by subsequently obtaining constraints on the amplitude [$\rho (x,t)$], phase [$\phi (x,t)$], EMF [$g_{2}(x,t)$], BMF [$g_{1}(x,t)$] nonliearities, and gain/loss [$\tau (t)$] of the considered system:

6 $$\begin{aligned} {[}\rho ^{2}(x,t)\eta _{x}(x,t)]_{x}=0, \;\;\;\;\;\; \eta _{t}(x,t)+\eta _{x}(x,t)\phi _{x}(x,t)=0, \end{aligned}$$

7 $$\begin{aligned} G_{1} \eta _{x}^{2}(x,t)-2 \rho (x,t) g_{1}(x,t)=0, \;\; G_{2} \eta _{x}^{2}(x,t)-2 \rho ^{2}(x,t) g_{2}(x,t)=0, \end{aligned}$$

8 $$\begin{aligned} \frac{\rho _{t}(x,t)}{\rho (x,t)}+\frac{1}{2 \rho ^{2}(x,t}[\rho ^{2}(x,t)\phi _{x}(x,t)]_{x}=\tau (t), \end{aligned}$$

with

9 $$\begin{aligned} \frac{\rho _{xx}(x,t)}{2\rho (x,t)}-\frac{\phi _{x}^{2}(x,t)}{2}-\phi _{t}(x,t)-\frac{1}{2} \mu \eta _{x}^{2}(x,t)-V(x,t)=0. \end{aligned}$$

In the above equations, the function denoted by the subscript signifies the partial differentiation of the function concerning the subscripted variable. Here, $\rho (x,t)$, $\phi (x,t)$, and $F[\eta (x,t)]$ represent the amplitude, phase, and traveling coordinate (similarity variable), respectively, all of which vary with space and time. Further, $\mu$, is the eigenvalue of the Eq. (5). The set of Eqs. (6)–(8) can be solved consistently to obtain:

10 $$\begin{aligned} \rho (x,t)= \sqrt{\frac{c(t)}{\eta _{x}(x,t)}}, \;\; \phi _{x,t}=-\frac{\eta _{t}(x,t)}{\eta _{x}(x,t)}, \;\; g_{1}(x,t) = G_{1} \frac{\eta _{x}^{5/2}(x,t)}{2 \sqrt{c(t)}}, \;\; g_{2}(x,t) = G_{2} \frac{\eta _{x}^{3}(x,t)}{2 c(t)}, \end{aligned}$$

where *c*(*t*) is the constant of integration, and $G_1$, $G_2$ represents the the strength of BMF, and EMF interactions, respectively. It is evident from the Eq. (10) that the form of amplitude, phase, MF, and BMF nonlinearities is directly dependent on the form of $\eta (x,t)$, which will be determined by solving the consistency Eq. (9). For that purpose, we substitute the trap expression from Eq. (1) into the consistency Eq. (9) and choose $\eta (x,t)= f[\eta (x,t)]=\int _{0}^{\eta } exp[\beta (t) \cos (l \eta )] \partial \eta$. Using this, we obtain the exact analytical form of the amplitude, phase, and nonlinearities:

11 $$\begin{aligned} \rho (x,t)= & \sqrt{\frac{c(t)}{ exp[\beta (t) \cos (l \eta )]}},\;\; \phi (x,t)=-\delta _{t}(t)x+ \left[ \frac{\beta ^{2}(t) k^{2}}{16}-\frac{1}{2}(\delta _{t}(t))^{2}\right] t,\;\; \tau (t)=\frac{1}{2}\frac{c_{t}(t)}{c(t)} \end{aligned}$$

12 $$\begin{aligned} g_{1}(x,t)= & \frac{G_{1} }{2 c(t)} exp[\beta (t) \cos (l \eta )]^{\frac{5}{2}},\;\; g_{2}(x,t)=\frac{G_{2}}{2 c(t)}exp[\beta (t) \cos (l \eta )]^{3}, \end{aligned}$$

such that

13 $$\begin{aligned} V_{1}(t)=\delta _{tt}(t), \;\; V_{2}=-\frac{\beta ^{2}(t) l^{2}}{16}, \;\;V_{3}=\frac{\beta (t) l^{2}}{4}, \;\; V_{3}^{\prime }=\left[ \frac{\beta (t) l^{2}}{4}+\mu \beta (t) \right] , \;\; V_{4}=\frac{\mu }{2}, \end{aligned}$$

where $\eta (x,t)=x-\delta (t)$ and $\delta (t)$ represents the center of mass coordinate of the droplet which is non-trivially connected with EMF, BMF, and BOL trap.

Finally, Eq. (5) represents the evolution of the droplets, for which an explicit solution can be formulated as: $F[\eta ]=\frac{3 (\mu /G_{1}) }{1+\sqrt{1-\frac{\mu }{\mu _{0}} \frac{ G_{2}}{ G_{1}^{2}} } \cosh (\sqrt{-\mu }\eta )}$ with $\mu _{0}=-2/9$, $E<0$, $G_1<0$, and $G_2>0$^29,38^. Utilizing this and Eq. (11), we write the complete solution of the Eq. (3):

14 $$\begin{aligned} \psi (x,t)=\sqrt{\frac{c(t)}{ exp[\beta (t) \cos (l \eta )]}} \frac{\frac{3 \mu }{G_{1}} \times exp\left[ i \{ -\delta _{t}(t)x+ \left[ \frac{\beta ^{2}(t)l^{2}}{16}-\frac{1}{2}(\delta _{t}(t))^{2}\right] t \} \right] }{1+\sqrt{1-\frac{\mu }{\mu _{0}} \frac{ G_{2}}{ G_{1}^{2}} } \cosh (\sqrt{-\mu } ( \int _{0}^{\eta } exp[\beta (t) \cos (l \eta )]))}. \end{aligned}$$

Thus, it is worth indicating that by changing the form of $\delta (t)$ one can introduce different driving fields in the considered system and modulation of $\beta (t)$ results in temporal variation of BOL’s potential depth. Here, we constructed a large family of exact analytical solutions of the 1D eGPE for the considered tilted and driven BOL trap configuration.

In the following section, we examine the dynamics of QDs using the precise solution (14) of the 1D eGPE under the influence of temporally changing repulsive cubic EMF and attractive quadratic BMF interactions within dc and ac driving fields. Utilizing Eq. (13), we explore the dynamics of the droplets for following experimentally pertinent forms, selecting appropriate driving frequencies $\delta (t)$ and potential depths $\beta (t)$.

**Table 1:** Various confinement forms by tuning $\delta (t)$, and $\beta (t)$.

Trap form	$\delta (t)$	$\beta (t)$	Trap expression
Free space	Constant	0	*V*(*x*, *t*) = constant
Linear dc field driven BOL	$$\delta$$$$\times$$ t	$$\beta$$	$$V(x,t) \simeq -\frac{\beta ^{2}l^{2}}{16} \cos [2l\{x - \delta t \}] + \left[ \frac{\beta l^{2}}{4}+\mu \beta \right] \cos [l\{x- \delta t\}]$$
Tilted and periodic ac field driven BOL	$$\delta$$$$\cos (t)$$	$$\beta$$	$$V(x,t) \simeq \delta \cos (t) x -\frac{\beta ^{2}l^{2}}{16} \cos [2l\{x - \delta \cos (t) \}] + \left[ \frac{\beta l^{2}}{4}+\mu \beta \right] \cos [l\{x- \delta \cos (t)\}]$$
Tilted and bi-periodic ac field driven BOL	$\delta _{1} \cos (t)+\delta _{2} cos(2t)$	$\beta$	$V(x,t) \simeq (\delta _{1} \cos (t)+\delta _{2} cos(2t))x-\frac{\beta ^{2}l^{2}}{16} \cos [2l\{x -\delta _{1} \cos (t)-\delta _{2} cos(2t) \}] + \left[ \frac{\beta l^{2}}{4}+\mu \beta \right] \cos [l\{x -\delta _{1} \cos (t)-\delta _{2} cos(2t)\}]$
Tilted and periodic ac field driven BOL with perturbed potential depth	$\delta \cos (t)$	$\beta (1+\alpha \sin (p t))$	$V(x,t) \simeq \delta \cos (t) x -\frac{[\beta (1+\alpha \sin (p t))]^{2}l^{2}}{16} \cos [2l\{x - \delta \cos (t) \}] + \left[ \frac{[\beta (1+\alpha \sin (p t))] l^{2}}{4}+\mu \beta \right] \cos [l\{x- \delta \cos (t)\}]$

Where $\delta$, $\beta$, *p*, $\delta _{1}$, $\delta _{2}$ are constants. Depending on the requirements of the specific physical scenario, one can select an appropriate form for $\delta (t)$ and $\beta (t)$ to regulate the driving fields and potential depths of the resulting traps. In the subsequent sections, we illustrate the pattern formation on QDs in the above-mentioned trap configurations given in Table 1. We illustrate the generation of various pattern morphologies on QDs by tuning the driving field and potential depth of chosen tilted and driven BOL confinement. Here, we take $(t)=c$ (constant) in Eq. (11), such that $\tau (t)=0$ leading to zero gain or loss of atoms in the considered system.

**Fig. 1:**
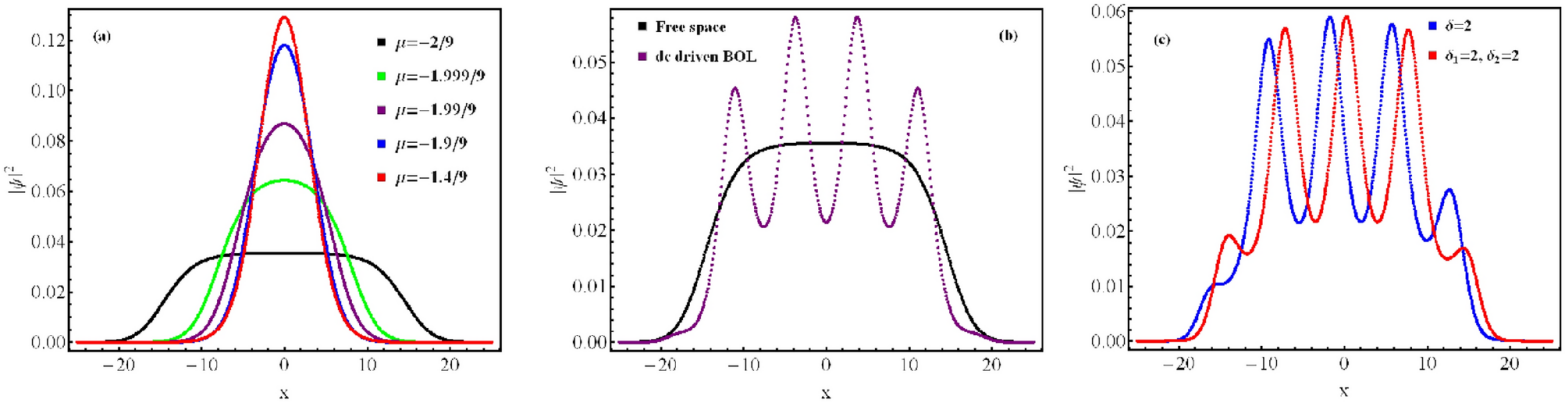
(**a**) Formation of QDs in free space with the variation of the $\mu$ akin to chemical potential of the system for $\mu =- 2/9$, 1.999/9, $- 1.99/9$ and $- 1.4/9$ along with $\delta =0$, $\beta =0$. (**b**) Comparison of the atomic condensate densities in presence of the dc driven BOL and free space with $\delta = 2$, $\beta (t)=\beta =0.5$ and $\mu =-2/9$ at $t=0$. (**c**) The condensate density profile in presence of periodic ($\delta =2$), and bi-periodic ($\delta _{1}=2$, $\delta _{2}=2$) driven BOL trap at $t=0$ with $\beta (t)=\beta =0.5$. In all the cases, the magnitude of physical parameters are: $c=1$, $l=0.84$, $G_{1}=- 1$, $G_{2}=0.999999$ and the spatial coordinate is scaled by the oscillator length.

First of all, from the constructed wavefunction solution (14) for the chosen potential (1), we illustrate the generation of QDs in free space by choosing $\beta (t)=0$, and $\delta =0$, such that $V(x)=$ constant. In this case, the wavefunction becomes: $\psi (x,t)= \frac{\sqrt{c} \frac{3 \mu }{G_{1}} }{1+\sqrt{1-\frac{\mu }{\mu _{0}} \frac{ G_{2}}{ G_{1}^{2}} } \cosh (\sqrt{-\mu } x)}$, and is identical to previously reported results for free space^38,43^. In the Fig. 1a, the formation of QDs is illustrated in free space with the variation of the $\mu$ akin to chemical potential of the system for $\mu =- 2/9$, 1.999/9, $- 1.99/9$ and $- 1.4/9$ along with $\delta =0$, $\beta =0$.

**Fig. 2:**
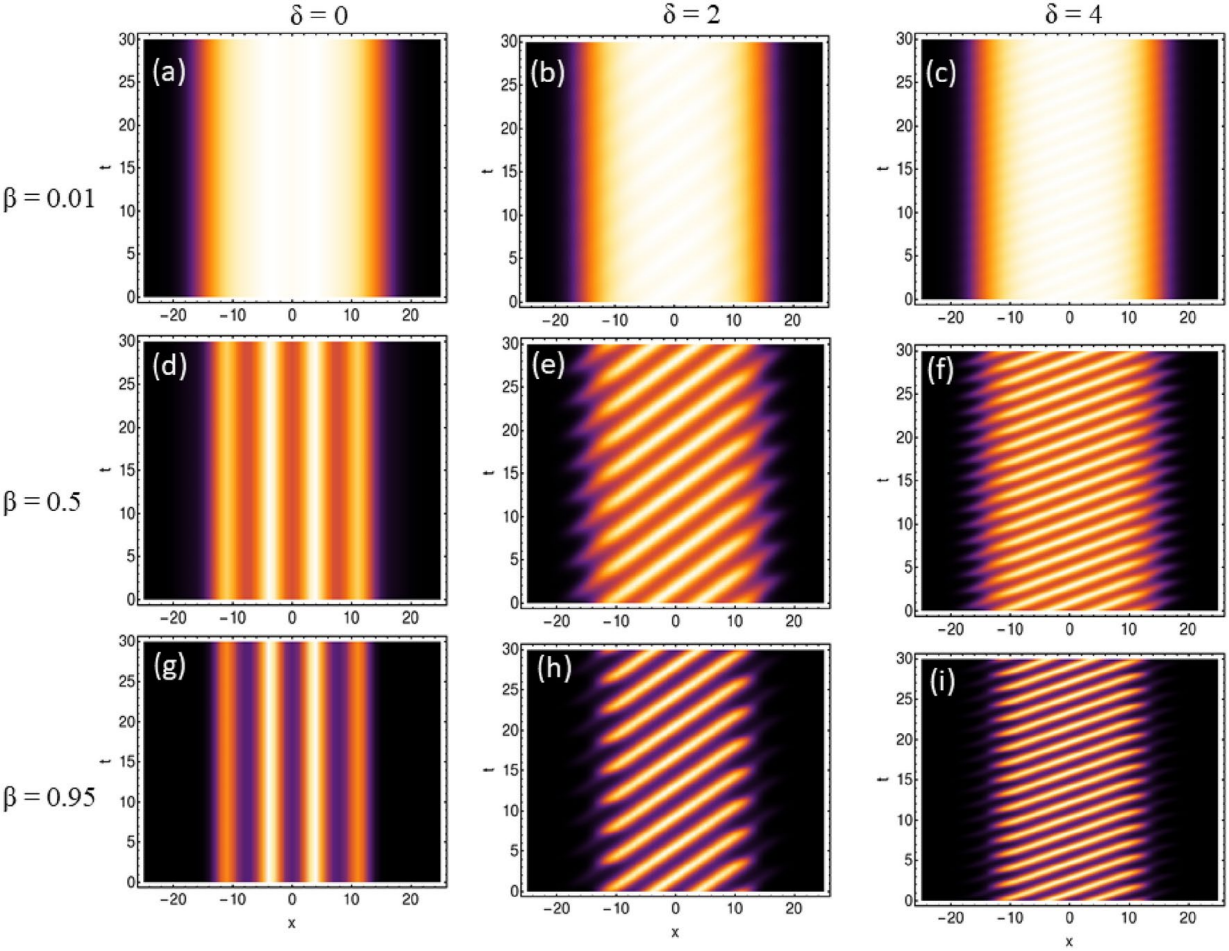
Formation of temporal stripe-like pattern under dc driving field ($- \delta t$): The profile of condensate patterns with the variation of the amplitude of BOL potential depth ($\beta$) and linear driving force strength ($\delta$). In this, the magnitude of physical parameters are: $c=1$, $l=0.84$, $\mu =-2/9$, $G_{1}=-1$, $G_{2}=0.999999$ along with $\beta =0.01$, 0.5, 0.95 and $\delta =0$ (absence of driving force), 2, 4. The spatial coordinate is scaled by the oscillator length.

### QDs in linear dc field driven BOL

In this section, we illustrate the stripe-like pattern on QDs in the temporal domain under the influence of dc shaking of BOL trap. For that purpose we take $\delta (t)=\delta t$ with $\delta \ne 0$ and $-1<\beta <1$ in the potential Eq. (2). Thus, the effective form of resultant potential is: $V(x,t) \simeq -\frac{\beta ^{2}l^{2}}{16} \cos [2l\{x - \delta t \}] + \left[ \frac{\beta l^{2}}{4}+\mu \beta \right] \cos [l\{x- \delta t\}].$ Here, the amplitude of BOL potential depth ($\beta$) and linear driving force strength ($\delta$) are two experimentally tunable trap parameters. The corresponding wavefunction solution can be written by using Eq. (14) as:

15 $$\begin{aligned} \psi (x,t)=\sqrt{\frac{c}{ exp[\beta \cos \{l (x- \delta t)\}]}} \frac{\frac{3 \mu }{G_{1}} \times exp\left[ i \{ -\delta x+ \left[ \frac{\beta ^{2}l^{2}}{16}-\frac{1}{2}(\delta )^{2}\right] t \} \right] }{1+\sqrt{1-\frac{\mu }{\mu _{0}} \frac{ G_{2}}{ G_{1}^{2}} } \cosh (\sqrt{-\mu } ( \int _{0}^{x} exp[\beta \cos \{l (x- \delta t)\}]))}, \end{aligned}$$

where $c(t)=c$ (constant) such that $\tau =0$ i.e. no gain or loss of condensate atoms in the system. Here, the form of BMF and EMF nonlinearities are $g_{1}(x,t)=\frac{G_{1} }{2 c} exp[\beta \cos \{l (x- \delta t)\}]^{\frac{5}{2}}$, $g_{2}(x,t)=\frac{G_{2}}{2 c}exp[\beta \cos \{l (x- \delta t)\}]^{3},$ respectively.

In comparison to free space, the periodic condensate patterns are formed due to the presence of dc driven BOL as evident from Fig. 1b. In Fig. 2a–i, we investigate the profile of condensate patterns with the variation of the amplitude of BOL potential depth ($$\beta$$) and linear driving force strength ($$\delta$$). In this, the magnitude of physical parameters are: $$c=1$$, $$l=0.84$$, $$\mu =-2/9$$, $$G_{1}=-1$$, $$G_{2}=0.999999$$ along with $$\beta =0.01$$, 0.5, 0.95 and $$\delta =0$$ (absence of driving force), 2, 4. It is evident from the figure that for $$\beta =0.01$$ and $$\delta =0$$, the system remains in the droplet phase as no distinct separation of atomic condensate density is observed (Fig. 2a). Further, $$\delta$$$$\rightarrow$$ 2 $$\rightarrow$$ 4 leads to the generation of visible stripe-like patterns on the QDs for $$\beta =0.01$$ (Fig. 2b,c). In the Fig. 2e,h, these patterns become dominant with $$\beta$$$$\rightarrow$$ 0.5 $$\rightarrow$$ 0.95 with $$\delta \ne$$ 0. The generation of these patterns is due to $$-\delta t$$ dc driving of BOL confinement. Additionally, we observe that for $$\delta =2$$, $$\beta =0.5$$, the width of the stripe-like pattern is large but less in number whereas for the same $$\beta$$ with $$\delta \rightarrow 2\rightarrow 4$$ leads to a decrease in width and enhancement in the number of stripe-like patterns (Fig. 2e–f,h–i. Thus, the driving strength ($$\delta$$) of the BOL trap is controlling the width and periodicity of stripe-like patterns on the QDs. It is important to note here that an increase in the magnitude of $$\beta \rightarrow 0.01 \rightarrow 0.5 \rightarrow 0.95$$ for $$\delta =0$$ (i.e. in absence of driving) case leads to fragmentation of droplet into gap solitons (Fig. 2a,d,g). This also leads to compression of atomic density width into gap solitons and Anderson-like localization of these solitons. This can be attributed to the fact that an increase in $$\beta$$ leads to an increase in the potential depths of the BOL trap and this leads to a decrease in the effective barrier heights of two neighboring BOL wells. This enhances the inter-site quantum tunneling of condensate atoms and leads to the observation of compression of condensate atomic density width and Anderson-like localization leading to formation of supersolid like structure. Thus, a non-trivial role of $$\beta$$ and $$\delta$$ is revealed which controls the rate of droplet-to-soliton transition and generation of temporal stripe-like patterns on QDs in the presence of disordered potential like BOL.

**Fig. 3:**
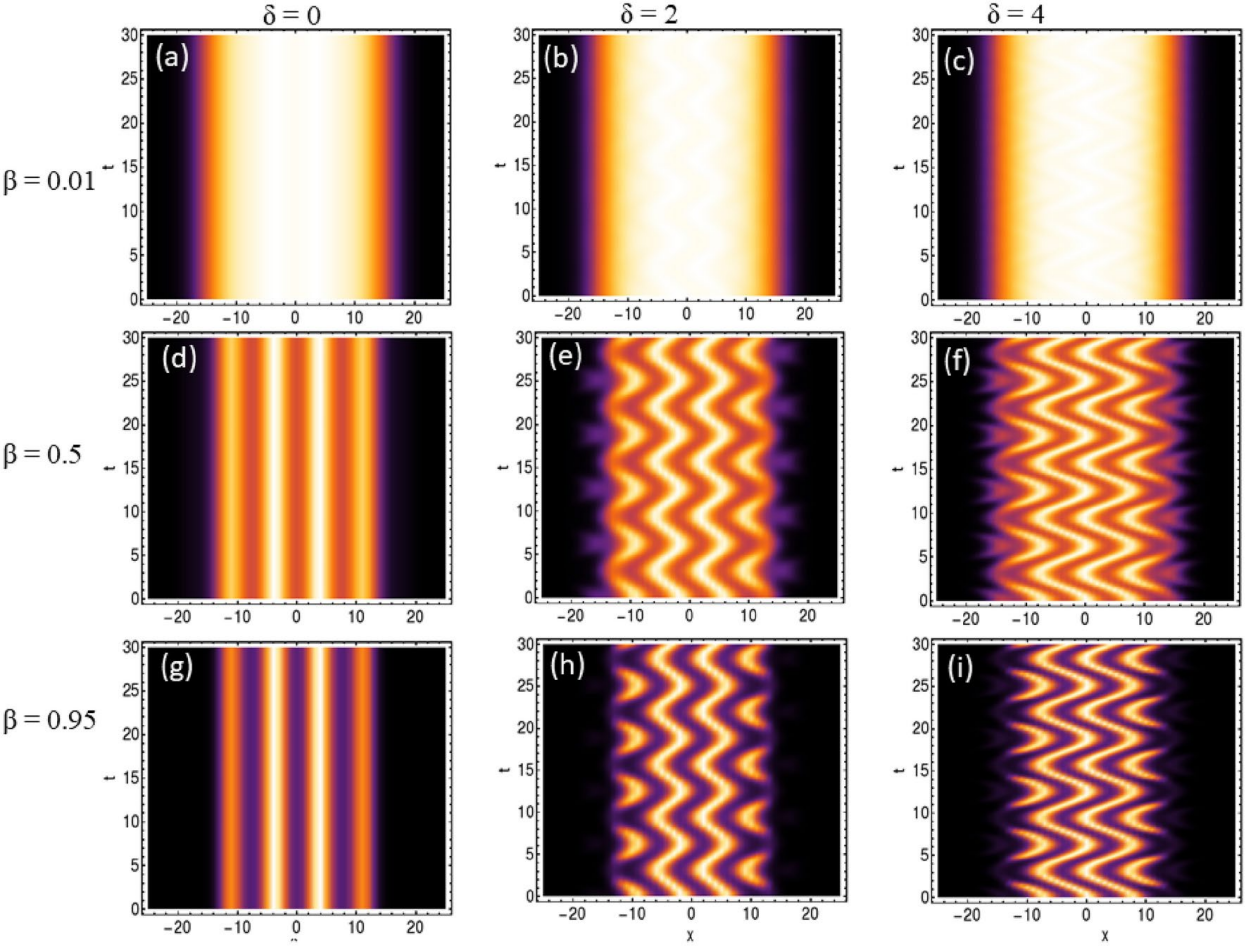
Generation of temporal periodic oscillating density pattern under ac driving field ($- \delta \cos (t)$): The profile of condensate patterns with the variation of the amplitude of BOL potential depth ($\beta$) and linear driving force strength ($\delta$). In this, the magnitude of physical parameters are: $c=1$, $l=0.84$, $\mu =-2/9$, $G_{1}=-1$, $G_{2}=0.999999$ along with $\beta =0.01$, 0.5, 0.95 and $\delta =0$ (absence of driving force), 2, 4. The spatial coordinate is scaled by the oscillator length.

### QDs in tilted and periodic ac field driven BOL

Different from the previous section, here we illustrate the temporal periodic oscillation of density waves on the QDs under the influence of temporal periodic driving of BOL trap. For that purpose we take $\delta (t)=-\delta \cos (t)$ with $\delta \ne 0$ and $-1<\beta <1$ in the potential Eq. (2). Thus, the effective form of resultant potential is: $V(x,t) \simeq \delta \cos (t) x -\frac{\beta ^{2}l^{2}}{16} \cos [2l\{x - \delta \cos (t) \}] + \left[ \frac{\beta l^{2}}{4}+\mu \beta \right] \cos [l\{x- \delta \cos (t)\}]$. Here, two experimentally adjustable trap parameters include the amplitude of BOL potential depth ($\beta$) and the strength of the linear driving force ($\delta$). The wavefunction solution corresponding to this can be expressed using Eq. (14) as:

16 $$\begin{aligned} \psi (x,t)=\sqrt{\frac{c}{ exp[\beta \cos \{l (x- \delta \cos (t))\}]}} \frac{\frac{3 \mu }{G_{1}} \times exp\left[ i \{ \delta \sin (t) x+ \left[ \frac{\beta ^{2}l^{2}}{16}-\frac{1}{2}(\delta )^{2}\right] t \} \right] }{1+\sqrt{1-\frac{\mu }{\mu _{0}} \frac{ G_{2}}{ G_{1}^{2}} } \cosh (\sqrt{-\mu } ( \int _{0}^{x} exp[\beta \cos \{l (x- \delta \cos (t))\}]))}. \end{aligned}$$

Similar to the previous case, we choose $$c(t)=c$$ (constant) such that $$\tau =0$$ i.e. no gain or loss of condensate atoms in the system. In this case, the BMF and EMF nonlinearities are: $$g_{1}(x,t)=\frac{G_{1} }{2 c} exp[\beta \cos \{l (x- \delta \cos (t))\}]^{\frac{5}{2}},$$$$g_{2}(x,t)=\frac{G_{2}}{2 c}exp[\beta \cos \{l (x- \delta \cos (t))\}]^{3},$$ respectively.

Using the wavefunction solution provided in Eq. (16), we explore the emergence of patterns on QDs under temporal periodic driving of the BOL trap, as shown in Fig. 3a–i. Similar to the previous scenario, in Fig. 3a,d,g, where no driving field is present ($\delta =0$) and $\beta$ varies from $0.01 \rightarrow 0.5 \rightarrow 0.95$, the pattern transitions from a droplet phase to gap solitons. Additionally, a decrease in the width of the atomic condensate density and Anderson-like localization is observed with increasing $\beta$ magnitude. Subsequently, when $\delta$ varies from $0 \rightarrow 2 \rightarrow 4$ for $\beta =0.01$, temporal periodic oscillations of density are visible on the QDs phase in Fig. 3b,c. These temporal periodic patterns become more pronounced with increasing $\beta$ magnitude ($0.5 \rightarrow 0.95$), as evident from Fig. 3e,h. Furthermore, an increase in the magnitude of $\delta$ from $2 \rightarrow 4$ results in the amplification of temporal density oscillation amplitude, as depicted in Fig. 3e,f,h,i. Hence, by adjusting the magnitude of $\beta$, the brightness of the formed patterns can be controlled, while $\delta$ governs the amplitude of the temporally formed density patterns.

**Fig. 4:**
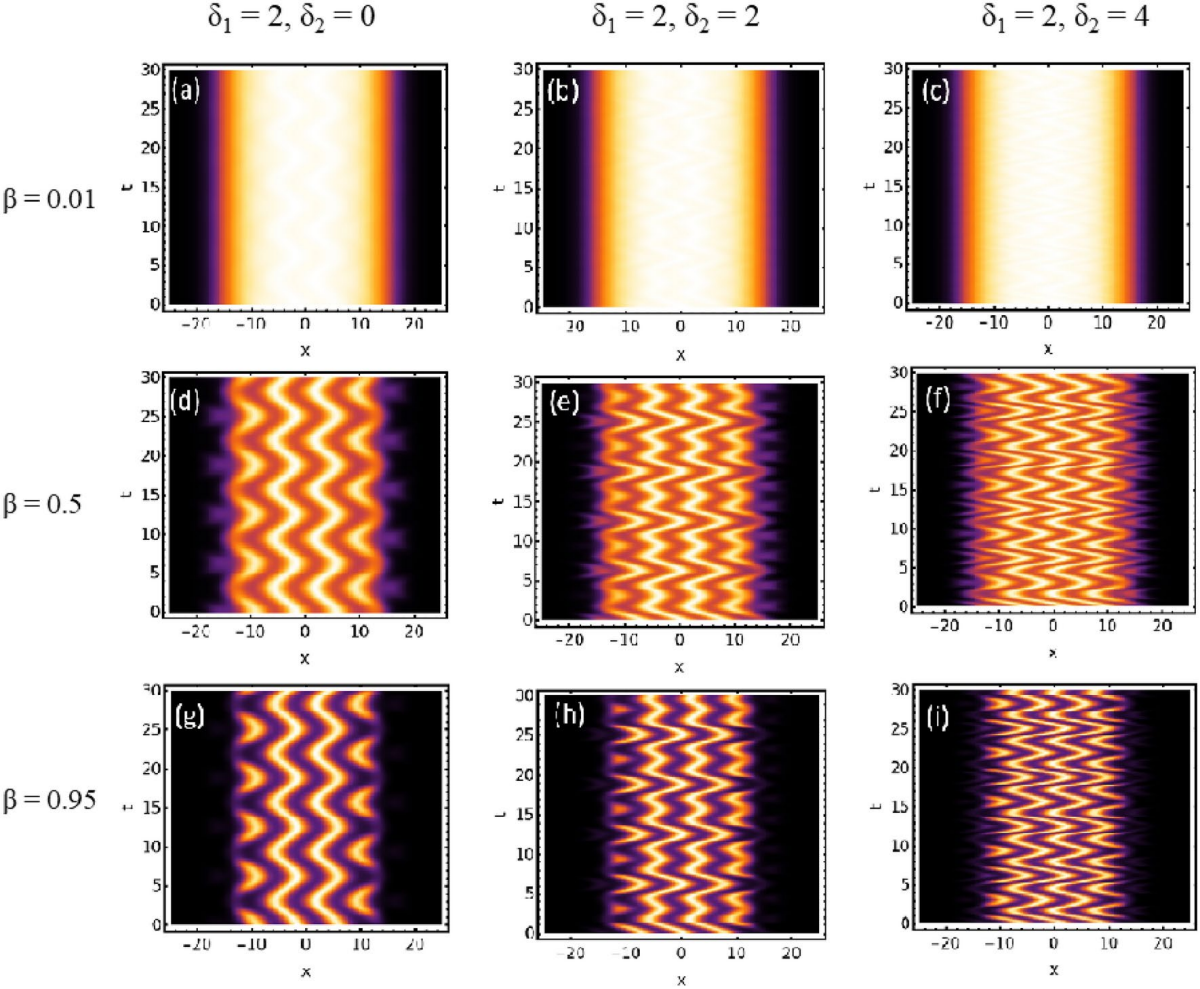
Generation of temporal bi-periodic oscillating density pattern under ac driving field $(-\delta _{1} \cos (t)-\delta _{2} cos(2t))$: The profile of condensate patterns with the variation of the amplitude of BOL potential depth (β) and linear driving force strength$(\delta _{1},\delta _{2})$. In this, the magnitude of physical parameters are: c = 1, l = 0.84, μ = −2/9, G1 = −1, G2 = 0.999999 along with β = 0.01, 0.5, 0.95, $\delta _{1}=2, and \, \delta _{2} = 0$ (absence of bi-periodic drivingforce), 2, 4. The spatial coordinate is scaled by the oscillator length.

### QDs in tilted and bi-periodic ac field driven BOL

In this section, we consider a temporal bi-periodic driving of BOL confinement i.e. $$\delta (t) = -\delta _{1} \cos (t)-\delta _{2} cos(2t)$$ with $$\delta _{1} \ne 0$$ and $$\delta _{2} \ne 0$$. For that purpose, we take the form of BMF and EMF nonlinearities are $$g_{1}(x,t)=\frac{G_{1} }{2 c} exp[\beta \cos \{l (x -\delta _{1} \cos (t)-\delta _{2} cos(2t))\}]^{\frac{5}{2}}$$, $$g_{2}(x,t)=\frac{G_{2}}{2 c}exp[\beta \cos \{l (x -\delta _{1} \cos (t)-\delta _{2} cos(2t))\}]^{3},$$ respectively. This results in the effective form of resultant potential from Eq. (2): $$V(x,t) \,\simeq\, (\delta _{1} \cos (t)+\delta _{2} cos(2t))x-\,\frac{\beta ^{2}l^{2}}{16} \cos [2l\{x -\delta _{1} \cos (t)\,-\delta _{2} cos(2t) \}] \,+ \left[ \frac{\beta l^{2}}{4}+\mu \beta \right] \cos [l\{x -\delta _{1} \cos (t)-$$$$-\delta _{2} cos(2t)\}]$$. Here, like previous cases, we choose zero gain or loss of atoms in the system i.e. $$c(t) = c$$ (constant) and $$-1<\beta <1$$. Thus, the corresponding wavefunction solution can be written by using Eq. (14) as:

17 $$\begin{aligned} \psi (x,t)&=\sqrt{\frac{c}{ exp[\beta \cos \{l (x -\delta _{1} \cos (t)-\delta _{2} cos(2t))\}]}}\nonumber \\&\quad \times \frac{\frac{3 \mu }{G_{1}} \times exp\left[ i \{ -(-\delta _{1} \cos (t)-4\delta _{2}\cos (2t)) x+ \left[ \frac{\beta ^{2}l^{2}}{16}-\frac{1}{2}(\delta )^{2}\right] t \} \right] }{1+\sqrt{1-\frac{\mu }{\mu _{0}} \frac{ G_{2}}{ G_{1}^{2}} } \cosh (\sqrt{-\mu } ( \int _{0}^{x} exp[\beta \cos \{l (x -\delta _{1} \cos (t)-\delta _{2} cos(2t))\}]))}. \end{aligned}$$

**Fig. 5:**
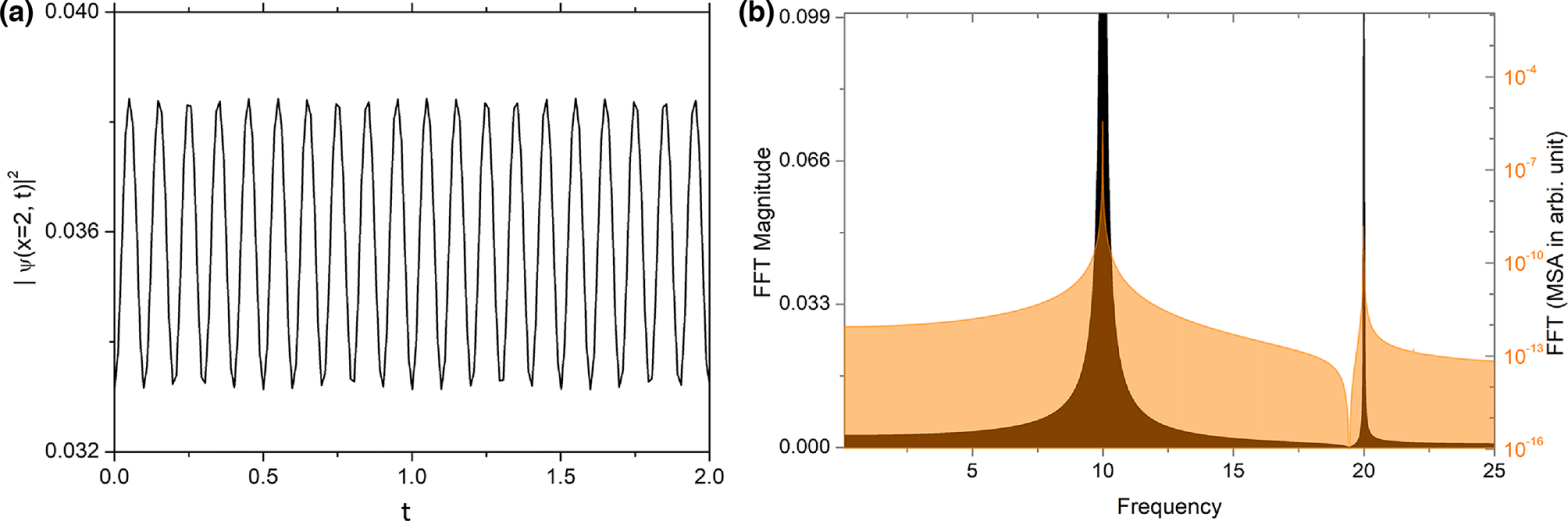
In (**a**) we have depicted droplets density variation at $x=2$ in presence of single frequency ($\delta (t) =-\delta \cos (q t)$) driven and tilted BOL trap with $\delta =1$, $q=10$. (**b**) This figure represents the frequency spectrum corresponding to the density oscillation of the droplets. Along x-axis the frequency which is scaled by trap frequency and left side y-axis presents FFT in arbitrary unit whereas FFT in mean square amplitude is represented in the right side y-axis. We see frequency spikes are present at 10(*q*) and 20(2*q*) which are the harmonics of *q*. In this, the magnitude of physical parameters are: $c=1$, $l=0.84$, $\mu =-2/9$, $G_{1}=-1$ and $G_{2}=0.999999$.

**Fig. 6:**
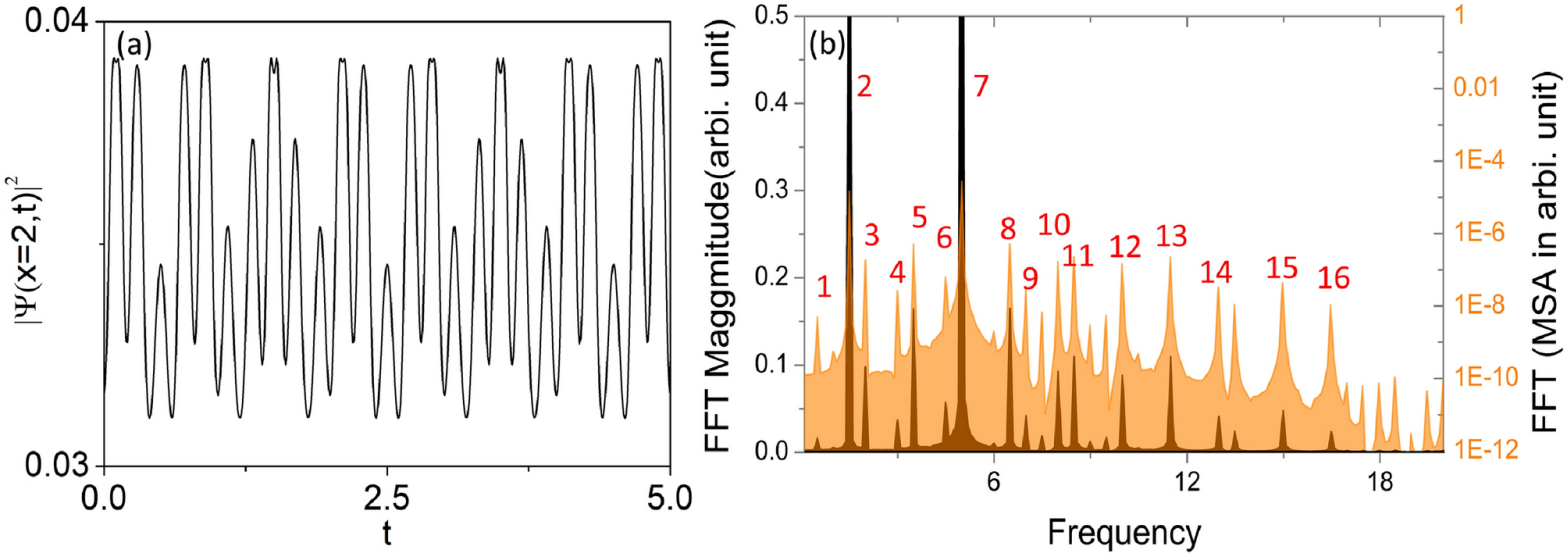
In (**a**) we have depicted droplets density variation at $x=2$ in presence of temporal periodic perturbation to the depth of the tilted and driven BOL. (**b**) This figure represented the frequency spectrum corresponding to the density oscillation of the droplets. Along x-axis the frequency which is scaled by trap frequency and left side y-axis presents FFT in arbitrary unit whereas FFT in mean square amplitude is represented in the right side y-axis. A few frequency spikes which we are labelled: $1=0.5 (q-3p)$; $2=1.5(p)$; $3=2(q-2p)$; $4=3(2p)$; $5=3.5(q-p)$; $6=4.5(3p)$; $7=5(q)$; $8=6.5(p+q)$; $9=7(2q-p)$; $10=8(2p+q)$; $11=8.5(2q-p)$; $12=10(2q)$; $13=11.5(p+2q)$; $14=13(2p+2q)$; $15=15(3q)$; $16=16.5(p+3q)$. In this, the magnitude of physical parameters are: $c=1$, $l=0.84$, $\mu =-2/9$, $G_{1}=-1$, $G_{2}=0.999999$ along with $\delta =1$, $q=5$.

In order to understand the impact of $\delta _{2} \cos (2t)$ on the pattern formation in comparison to the previous periodic driving case, we choose $\delta _{1}=2$ which is equivalent to Fig. 3b,e,h for $\beta =0.01$, 0.5, and 0.95 cases. Further, Fig. 1c shows the condensate density profile in presence of periodic ($\delta =2$), and bi-periodic ($\delta _{1}=2$, $\delta _{2}=2$) driven BOL trap at $t=0$ with $\beta (t)=\beta =0.5$. In Fig. 4a–c, with $\delta _{2} \rightarrow 0 \rightarrow 2 \rightarrow 4$, the temporal bi-periodic oscillation of density wave is observed. Like the previous case, these patterns become prominent with $\beta$ magnitude changing from $0.01 \rightarrow 0.5 \rightarrow 0.95$ (Fig. 4d,e,g–i). Figure 4f,i interestingly illustrates the increase in the depth of the smaller density depth in the bi-periodic pattern as $\delta$ changes from $2 \rightarrow 4$.

### Higher harmonics generation in QDs in tilted and periodic ac field driven BOL

Above, we thoroughly explored the density profile and dynamics of the droplets in the presence of various types of moving BOL configurations, which proved to be quite intriguing. However, it is worth noting that in all cases discussed, the BOL depth, proportional to the $\beta$ parameter, remained time-independent or constant. Now, to further elucidate our analysis, we have extended our dynamic investigation by introducing a perturbation to the potential depth of the tilted and driven BOL by tuning $\beta (t)$. Here, the potential profile is: $V(x,t) \simeq \delta q^{2} \cos (t) x -\frac{\beta ^{2}l^{2}}{16} \cos [2l\{x - \delta \cos ( q t) \}] + \left[ \frac{\beta l^{2}}{4}+\mu \beta \right] \cos [l\{x- \delta \cos (q t)\}]$ and the corresponding wavefunction can be written using Eq. (14).

In the Fig. 5a, we have presented the density oscillation of the droplets at $x=2$ with the time when BOL is moving to-fro sinusoidal form with a frequency *q* with constant $\beta$. The parameters which are considered $\beta =0.1$, $\delta =1$ which is perturbation amplitude and $q=10$ is the frequency of the ac driven field. Upon performing Fast Fourier transformation (FFT) using Origin software, we observe two frequencies in Fig. 5b, one frequency due to moving of the BOL is $q=10$ and other one is the first higher harmonics at 20. This kind of higher harmonics generation only we can see in case of single frequency ac driven field. But, the scenarios will be more interesting when ac driven field and perturbation to the depth of the OL are simultaneously acting in the system.

#### Temporal periodic perturbation of chosen trap potential depth

Different from previous scenario, here we take the perturbation form: $$\beta (t)= \\ \beta (1+\alpha \sin (p t))$$ which results in the effective form of resultant potential as: $$V(x,t) \simeq \delta \cos (t) x  \\ \,-\,\frac{[\beta (1+\alpha \sin (p t))]^{2}l^{2}}{16} \cos [2l\{x \,-\, \delta \cos (q t) \}] + \left[ \frac{[\beta (1+\alpha \sin (p t))] l^{2}}{4}\,+\,\mu [\beta (1+\alpha \sin (p t))] \right]\cos [l\{x\,-\, $$$$\delta \cos (q t)\}]$$. Here, two experimentally adjustable trap parameters include the amplitude of BOL potential depth ($$\beta$$) and the strength of the linear driving force ($$\delta$$). The wavefunction solution corresponding to this can be expressed using Eq. (14) as:

18 $$\begin{aligned} \psi (x,t)= & \sqrt{\frac{c}{ exp[[\beta (1+\alpha \sin (p t))] \cos \{l (x- \delta \cos (q t))\}]}} \nonumber \\ & \quad \times \frac{\frac{3 \mu }{G_{1}} \times exp\left[ i \{ \delta \sin (t) x+ \left[ \frac{[\beta (1+\alpha \sin (p t))]^{2}l^{2}}{16}-\frac{1}{2}(\delta )^{2}\right] t \} \right] }{1+\sqrt{1-\frac{\mu }{\mu _{0}} \frac{ G_{2}}{ G_{1}^{2}} } \cosh (\sqrt{-\mu } ( \int _{0}^{x} exp[(\beta (1+\alpha \sin (p t))) \cos \{l (x- \delta \cos (q t))\}]))}. \end{aligned}$$

In Fig. 6a, we depict the density oscillation of the droplets at $x=2$ over time, where the tilted and driven BOL confinement not only undergoes motion but also experiences sinusoidal perturbation with frequency *p*. The parameters considered are $\beta =0.1$, $\delta =1$, $q=5$ (BOL moving frequency), $\alpha =0.4$ (perturbation amplitude), and $p=1.5$ (perturbation frequency). Notably, the oscillation comprises many frequencies. Specifically, there is one fundamental frequency attributed to the perturbation frequency $p=1.5$, as anticipated in the dynamics. Upon performing FFT using Origin software, we observe the presence of many frequencies alongside fundamental frequency 1.5 (refer to Fig. 6b). Two frequencies, one frequency due to moving of the BOL is $q=5$ and other one is due perturbation $p=1.5$ are two major frequencies. Rest of all higher harmonics and combination of frequencies (refer to Fig. 6b). A few frequency spikes which we are labelled are given below in Table 2. The generation of higher harmonics and combination of frequencies on QDs in the chosen trap configuration is the clear signature of the nonlinear dynamical system.

**Table 2:** Generated higher and combinational frequencies in periodically temporally perturbed potential depths of tilted and driven BOL for $p=1.5$ and $q=5$.

Label	Frequency	Combination of frequencies
1	0.5	$q-3p$
2	1.5	*p*
3	2	$q-2p$
4	3	2*p*
5	3.5	$q-p$
6	4.5	3*p*
7	5	*q*
8	6.5	$p+q$
9	7	$2q-p$
10	8	$2p+q$
11	8.5	$2q-p$
12	10	2*q*
13	11.5	$p+2q$
14	13	$2p+2q$
15	15	3*q*

**Fig. 7:**
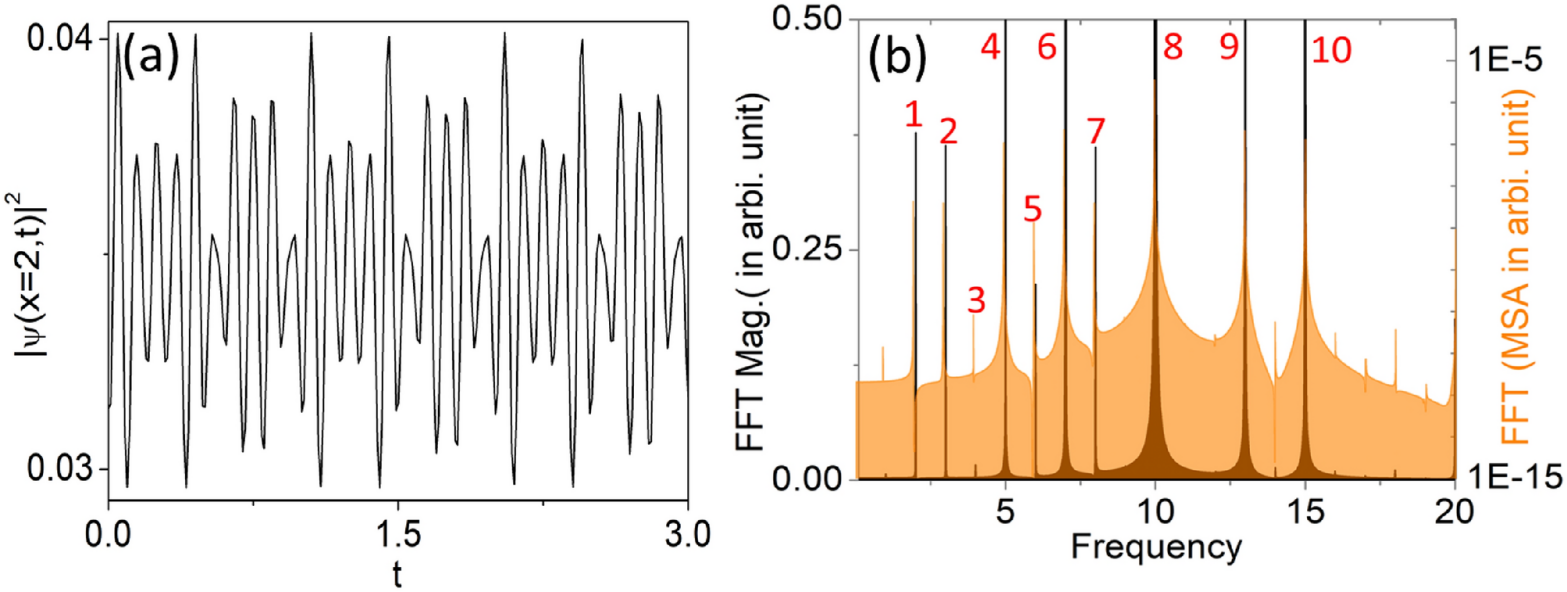
Generation of higher harmonics in QDs under temporal bi-periodic perturbation of depth of tilted and driven BOL: (**a**) The profile of condensate patterns at $$x=2$$ with the chosen form of $$\beta (t)= \beta (1+\alpha _{1} \sin (p_{1} t)+\alpha _{2} \sin (p_{2} t))$$, with $$\beta =0.1$$, $$\alpha _{1}=0.5$$, $$\alpha _{2}=0.4$$, $$q=10$$, $$p_{1}=3$$ and $$p_{2}=5$$. (**b**) Represents the frequency spectrum corresponding to the density oscillation of the droplets. Along x-axis the frequency which is scaled by the chosen trap frequency and left side y-axis presents FFT in arbitrary unit whereas FFT in mean square amplitude is represented in the right side y-axis. A few frequency spikes which we are labelled: $$1=2(p_{2}- p_{1})$$; $$2=3(p_{1})$$; $$3=4(p_{1}/2+ p_{2}/2)$$; $$4=5(p_{2})$$ or $$q-p_{2})$$; $$5=6(2p_{1})$$; $$6=7(q-p_{1})$$; $$7=8(p_{2}+ p_{1})$$; $$8=10(q)$$; $$9=13(q+p_{1})$$; $$10=15(q+p_{2})$$. In this, the magnitude of physical parameters are: $$c=1$$, $$l=0.84$$, $$\mu =-2/9$$, $$G_{1}=-1$$, $$G_{2}=0.999999$$ along with $$\delta =1$$.

#### Temporal bi-periodic perturbation of chosen trap potential depth

Next, we consider the case of temporal bi-periodic perturbation to the depth of the tilted and driven BOL by taking the form of $\beta (t)= \beta (1+\alpha _{1}\sin (p_{1} t)+\alpha _{2} \sin (p_{2} t))$ i.e. bi-periodic temporal perturbation with $\delta (t)=-\delta \cos (q t)$, and $q=10$.

Similar to previous cases, in the Fig. 7a, we present the density oscillation of the droplets at ($$x=2$$) over time, with the BOL not only moving but also having its amplitude perturbed in a sinusoidal form with two frequencies, $$p_{1}$$ and $$p_{2}$$ with $$\beta (t) = \beta (1 + \alpha _{1} \sin (p_{1} t + \alpha _{2} \sin (p_{2} t))$$. The parameters considered are $$\beta = 0.1$$, $$\alpha _{1} = 0.5$$, and $$\alpha _{2} = 0.4$$ as perturbation amplitudes, with $$p_{1} = 3$$ and $$p_{2} = 5$$ being the perturbation frequencies. Interestingly, the oscillation includes more than one frequency. Additionally, there is a frequency $$q = 10$$ due to the movement of the BOL. The Fig. 7b represents the frequency spectrum corresponding to the density oscillation of the droplets. The x-axis shows the frequency scaled by the trap frequency. The left y-axis presents the FFT in arbitrary units, while the right y-axis represents the FFT in mean square amplitude. Here, in Fig. 7b, the present frequencies are shown with labels, displaying several frequency spikes of varying heights. We have labeled a few spikes to demonstrate that all frequencies are combinations of harmonics and perturbation frequencies. A few labeled frequency spikes include: $$1=2(p_{2}- p_{1})$$; $$2=3(p_{1})$$; $$3=4(p_{1}/2+ p_{2}/2)$$; $$4=5(p_{2}$$ or $$q-p_{2})$$; $$5=6(2p_{1})$$; $$6=7(q-p_{1}$$; $$7=8(p_{2}+ p_{1})$$; $$8=10(q)$$; $$9=13(q+p_{1})$$; and $$10=15(q+p_{2})$$. Consequently, by precisely controlling the perturbation frequency and the frequency of the driven BOL, we can generate our desired frequency comb on QDs.

### Stability analysis

To enhance the realism of our analytical model, we perform stability analysis of the pattern formation in the time evolution of a binary mixture of BEC forming QDs. In realistic experimental settings, some degree of unavoidable noise is always present, whether from the system itself or its surroundings, and this noise can take various forms, including random fluctuations.

Specifically, we considered two scenarios: (i) stability against the noise added to the wavefunction, where a random white noise $R_{w}$ of amplitude 10 % of the maximum density is added and the BEC is allowed to evolve; and (ii) stability against the noise added to the trap, where a random white noise $R_{w}$ of amplitude 15% of the potential maximum is added and then the BEC is allowed to evolve. We have used the corresponding notations as $\psi _{noisy}(x; t= 1) = \psi (x; t = 1) + R_{w}$ and $V_{noisy}(x, t) = V(x,t) + R_{w}$.

**Fig. 8:**
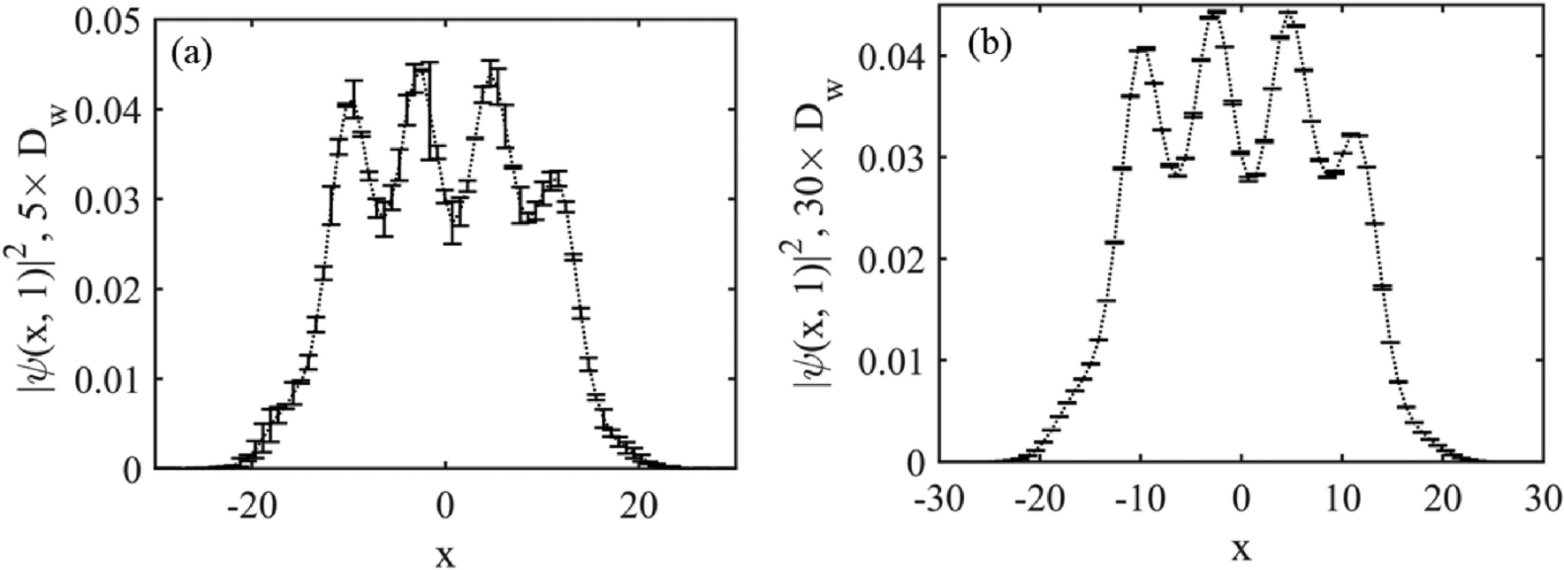
In this figure we have depicted structural stability of the QD. (**a,b**) Represents density deviation when noise is added to the initial sate and to the external confinement, respectively. Deviation $D_{w}$ is presented with error bar at different positions. Here, $\beta =0.1$, $l=0.84$, $\mu =-0.22$, $G_{1}=-1$, $G_{2}=-0.9999$ and $\delta =1$.

We evaluate the noisy condensate density and its deviation from the original density ($D_{w}$). It has been observed that for the given noise $R_{w}$, the deviation in the condensate density always remains less than 2.5 % of the density maxima for the first case, and the same for the second case becomes less than 1% as shown in Fig. 8.

The stability analyses have been performed for the case when $\beta =0.1$, $l=0.84$, $\mu =-0.22$, $G_{1}=-1$, $G_{2}=- 0.999$ and $\delta =1$ for instance and is depicted in Fig. 8. We have carried out the numerical stability analysis by solving the 1D-eGPE given in Eq. (3) using the split-step Fourier method in MatLab. The condensate density is observed for 10,000 time iterations with properly chosen space and time steps, $dx =0.078$ and $dt=0.001$, respectively. As the deviation is quite insignificant compared to the peak value of the condensate density, we have scaled them with appropriate factors while illustrating. The noisy density at time ($t=10$) after 10,000 iterations retains its shape, which implies that our analytical solutions are sufficiently stable for both kinds of noise inclusions as displayed in Fig. 8.

## Summary

Exploring pattern formation and dynamics in quantum fluids, particularly QDs, remains relatively uncharted territory, with many unanswered questions. This study delves into the generation of diverse patterns within a one-dimensional binary BEC mixture forming QDs under the influence of a tilted and driven BOL trap. By developing an exact analytical model, we identify the wavefunction’s precise form, phase, as well as the effects of cubic repulsive EMF and attractive quadratic BMF interactions, and gain/loss terms. Solving the corresponding 1D eGPE in the presence of the tilted and driven BOL allows us to explore the out-of-equilibrium dynamics of droplets subjected to dc and ac driven fields. The study showcases the emergence of distinct density wave patterns that spontaneously disrupt the lattice symmetry. Under the influence of a dc field, a stripe-like pattern emerges in the temporal domain, while ac fields lead to temporal periodic and bi-periodic oscillations of density waves. Notably, the width and period of these patterns directly correlate with the strength of the ac and dc fields. Additionally, by temporally modulating the BOL potential depth, a range of harmonics appears in the oscillations of the condensate density pattern. Utilizing FFT analysis, it is verified that these harmonics comprise multiple and combined frequencies, indicating potential utility in creating tailored frequency combs within QDs. For a better viability of our solution towards practical implementation, we have performed stability analyses under two scenarios: (i) stability against the noise (10 % of the maximum density) added to the wavefunction; and (ii) stability against the noise (15% of the potential maximum) added to the trap. The noisy densities in both the cases retain their shapes, implying our analytical solutions sufficiently stable.

## Methods

We start by examining a binary BEC system, where both components have equal masses and an identical number of atoms, subjected to BMF effects, specifically the LHY corrections for quantum fluctuations, within a selected tilted and driven BOL confinement. The equal equilibrium densities of the binary mixture components simplify and clarify the analysis of the results. In a 1D geometry, the system is governed by the following equation:

19 $$\begin{aligned} i \hbar \frac{\partial \psi _1}{\partial t} & + \frac{\hbar ^2}{2m}\frac{\partial ^2\psi _1}{\partial x^2} - (\chi _s(x, t) |\psi _1|^2 + \chi _c(x, t) |\psi _2|^2)\psi _1 \nonumber \\ & + \Lambda (x, t) (|\psi _1|^2+|\psi _2|^2)^{1/2}\psi _1-v(x, t) \psi _1 -\tau _{1}(t) \psi _1=0, \end{aligned}$$

20 $$\begin{aligned} i \hbar \frac{\partial \psi _2}{\partial t} & + \frac{\hbar ^2}{2m}\frac{\partial ^2\psi _2}{\partial x^2} - (\chi _c (x, t) |\psi _1|^2 + \chi _s (x, t) |\psi _2|^2)\psi _2 \nonumber \\ & + \Lambda (x, t)(|\psi _1|^2+|\psi _2|^2)^{1/2}\psi _2 -v(x, t) \psi _2 -\tau _{1}(t) \psi _2=0. \end{aligned}$$

In this context, $\psi _1$ and $\psi _2$ denote the wavefunctions of the first and second components of the binary BEC mixture, respectively, while *v*(*x*, *t*) represents the applied external confinement. The interaction parameters are defined as $g_{11} = g_{22}\equiv g=2 \hbar ^{2} a_{s}(x, t)/(m a^{2}_{\perp })$, and $g_{c}= g_{12}$ where $a_{\perp }$, and $a_{s}(x, t)$ represents the atomic scattering lengths for both inter- and intra-component interactions. In these equations, $\chi _s (x, t)=(g_{c}+3g)/2$ denotes the self-interaction coefficients, while $\chi _c (x, t)=(g_{c}-g)/2$ describes the cross-interaction coefficients, with $\Lambda (x, t)=\sqrt{m}g^{3/2}/(\pi \hbar )$^42,59^. The sign and magnitude of $\chi _c (x, t)$ and $\Lambda (x, t)$ depend on the scattering lengths for inter- and intra-component atomic interactions. Experimentally, these scattering lengths can be adjusted using the Feshbach resonance technique, allowing precise control over the intra- and inter-component interactions. Here, $\hbar$ is Planck’s constant, and *m* is the mass of the BEC atom. $\tau _{1}(t)$ is the time-dependent gain or loss of condensate atoms in the considered system. The positive values of $\tau _{1}(t)$ indicate scenarios where atoms are either introduced into the condensate from the thermal background or injected via a pumping mechanism from a reservoir. Conversely, negative values of $\tau _{1}(t)$ are associated with atoms leaving the condensate due to dipolar relaxation.

Considering the spinor components of the binary mixture as mutually symmetric $\psi _{1}=\psi _{2}=c_{0} \psi$ and substituting it in the above equations reduces the Hamiltonian to the following 1D dimensionless single eGPE^29^:

21 $$\begin{aligned} i\frac{ \partial \psi }{\partial t}= & - \frac{1}{2} \frac{ \partial ^2 \psi }{\partial x^2} - g_1(x,t) |\psi |\psi + g_2(x,t) |\psi |^2\psi + [V_{1}x\nonumber \\ & + V_{2}\cos [2l\{x - \delta (t)\}] + V_{3}^{\prime }\cos [l\{x - \delta (t)\}]\psi + i \tau (t) \psi . \end{aligned}$$

In this context, $\psi (x, t)$ represents the condensate wave function with $c_{0}=(2 \sqrt{g})^{3/2}/(\pi \xi (2|\delta g|)^{3/4}$, while $g_{1}(x, t)=\Lambda (x, t)$ and $g_{2}(x, t)=\chi _{s}(x, t) + \chi _{c}(x, t)$ correspond to the inter-atomic interaction strengths in the two-component BEC mixture, characterizing the BMF and EMF interactions, respectively. Using the similarity transformation technique, we assume an ansatz solution and link the 1D eGPE to a solvable PDE. This approach results in several constraints on the phase, MF, and BMF nonlinearities, expressed as PDEs. We then solve these equations to obtain the general form of the wavefunction solution and exact analytical form of the BOL potential depths.

## Data Availability

All data generated or analysed during this study are included in this published article. It can be reproduced by utilizing the form of wavefunction and considered trap profiles.

## References

[1] Cross, M. C. & Hohenberg, P. C. Pattern formation outside of equilibrium. *Rev. Mod. Phys.* **65**, 851 (1993).

[2] Zahn, H. P. et al. Formation of spontaneous density-wave patterns in dc driven lattices. *Phys. Rev. X* **12**, 021014 (2022).

[3] Kora, Y. & Boninsegni, M. Patterned supersolids in dipolar Bose systems. *J. Low Temp. Phys.* **197**, 337 (2019).

[4] Grebenev, S., Toennies, J. P. & Vilesov, A. F. Superfluidity within a small helium-4 cluster: The microscopic Andronikashvili experiment. *Science* **279**, 2083 (1998). 9516103 10.1126/science.279.5359.2083

[5] Arecchi, F., Boccaletti, S. & Ramazza, P. Pattern formation and competition in nonlinear optics. *Phys. Rep.* **318**, 1–83 (1999).

[6] Ahlers, G., Grossmann, S. & Lohse, D. Heat transfer and large scale dynamics in turbulent Rayleigh–Bénard convection. *Rev. Mod. Phys.* **81**, 503 (2009).

[7] Mouritsen, O. G. Pattern formation in condensed matter. *Int. J. Mod. Phys. B* **04**, 1925–1954 (1990).

[8] Turing, A. M. The chemical basis of morphogenesis. *Philos. Trans. R. Soc. Lond. B Biol. Sci.* **237**, 37–72 (1952).

[9] Maini, P. K., Painter, K. J. & Chau, H. N. P. Spatial pattern formation in chemical and biological systems. *J. Chem. Soc. Faraday Trans.* **93**, 3601–3610 (1997).

[10] von Hardenberg, J., Meron, E., Shachak, M. & Zarmi, Y. Diversity of vegetation patterns and desertification. *Phys. Rev. Lett.* **87**, 198101 (2001). 11690457 10.1103/PhysRevLett.87.198101

[11] Riedel, I. H., Kruse, K. & Howard, J. A self-organized vortex array of hydrodynamically entrained sperm cells. *Science* **309**, 300 (2005). 16002619 10.1126/science.1110329

[12] Liddle, A. R. & Lyth, D. H. *Cosmological Inflation and Large-Scale Structure* (Cambridge University Press, 2000).

[13] Strecker, K. E., Partridge, G. B., Truscott, A. G. & Hulet, R. G. Formation and propagation of matter-wave soliton trains. *Nature* **417**, 150–153 (2002). 11986621 10.1038/nature747

[14] Engels, P., Atherton, C. & Hoefer, M. A. Observation of Faraday Waves in a Bose–Einstein Condensate. *Phys. Rev. Lett.* **98**, 095301 (2007). 17359165 10.1103/PhysRevLett.98.095301

[15] Zhang, J. et al. Observation of a discrete time crystal. *Nature* **543**, 217–220 (2017). 28277505 10.1038/nature21413

[16] Sacha, K. & Zakrzewski, J. Time crystals: A review. *Rep. Prog. Phys.* **81**, 016401 (2018). 28885193 10.1088/1361-6633/aa8b38

[17] Hertkorn, J. et al. Pattern formation in quantum ferrofluids: From supersolids to superglasses. *Phys. Rev. Res.* **3**, 033125 (2021).

[18] Bera, J., Batin, A. Q., Ghosh, S., Malomed, B. & Roy, U. Generation of higher harmonics in dipolar Bose–Einstein condensates in periodically-modulated potentials. *Philos. Trans. R. Soc. A* **381**, 20220075 (2023). 10.1098/rsta.2022.0075 36842989

[19] Tanzi, L. et al. Supersolid symmetry breaking from compressional oscillations in a dipolar quantum gas. *Nature* **574**, 382 (2019). 31499510 10.1038/s41586-019-1568-6

[20] Böttcher, F. et al. Transient supersolid properties in an array of dipolar quantum droplets. *Phys. Rev. X* **9**, 011051 (2019).

[21] Zhang, Z., Yao, K. X., Feng, L., Hu, J. & Chin, C. Pattern formation in a driven Bose–Einstein condensate. *Nat. Phys.* **16**, 652 (2020).

[22] Böttcher, F. et al. New states of matter with fine-tuned interactions: Quantum droplets and dipolar supersolids. *Rep. Prog. Phys.* **84**, 012403 (2021). 33176284 10.1088/1361-6633/abc9ab

[23] Meinert, F. et al. Observation of many-body dynamics in long-range tunneling after a quantum quench. *Science* **344**, 1259 (2014). 24926015 10.1126/science.1248402

[24] Bernien, H. et al. Probing many-body dynamics on a 51-atom quantum simulator. *Nature* **551**, 579 (2017). 29189778 10.1038/nature24622

[25] Schmitt, M., Wenzel, M., Böttcher, F., Ferrier-Barbut, I. & Pfau, T. Self-bound droplets of a dilute magnetic quantum liquid. *Nature* **539**, 259 (2016). 27830811 10.1038/nature20126

[26] Cabrera, C. R. et al. Quantum liquid droplets in a mixture of Bose–Einstein condensates. *Science* **359**, 301 (2018). 29242233 10.1126/science.aao5686

[27] Barranco, M. et al. Helium nanodroplets: An overview. *J. Low Temp. Phys.* **142**(1), 1 (2006).

[28] Lee, T. D., Huang, K. & Yang, C. N. Eigenvalues and eigenfunctions of a Bose system of hard spheres and its low-temperature properties. *Phys. Rev.* **106**, 1135 (1957).

[29] Petrov, D. S. Quantum mechanical stabilization of a collapsing Bose–Bose mixture. *Phys. Rev. Lett.* **115**, 155302 (2015). 26550732 10.1103/PhysRevLett.115.155302

[30] Petrov, D. S. & Astrakharchik, G. E. Ultradilute lowdimensional liquids. *Phys. Rev. Lett.* **117**, 100401 (2016). 27636457 10.1103/PhysRevLett.117.100401

[31] Tylutki, M., Astrakharchik, G. E., Malomed, B. A. & Petrov, D. S. Collective excitations of a one-dimensional quantum droplet. *Phys. Rev. A* **101**, 051601 (2020).

[32] Luo, Z.-H., Pang, W., Liu, B., Li, Y.-Y. & Malomed, B. A. A new form of liquid matter: Quantum droplets. *Front. Phys.* **16**, 32201 (2021).

[33] Bulgac, A. Dilute quantum droplets. *Phys. Rev. Lett.* **89**(5), 050402 (2002). 12144429 10.1103/PhysRevLett.89.050402

[34] Ferrier-Barbut, I., Kadau, H., Schmitt, M., Wenzel, M. & Pfau, T. Observation of quantum droplets in a strongly dipolar Bose gas. *Phys. Rev. Lett.* **116**, 215301 (2016). 27284663 10.1103/PhysRevLett.116.215301

[35] Rakshit, D., Karpiuk, T., Brewczyk, M. & Gajda, M. Quantum Bose-Fermi droplets. *SciPost Phys.* **6**, 079 (2019).

[36] Smith, J. C., Baillie, D. & Blakie, P. B. Quantum droplet states of a binary magnetic gas. *Phys. Rev. Lett.* **126**, 025302 (2021). 33512210 10.1103/PhysRevLett.126.025302

[37] De Rosi, G., Astrakharchik, G. E. & Massignan, P. Thermal instability, evaporation, and thermodynamics of one-dimensional liquids in weakly interacting Bose–Bose mixtures. *Phys. Rev. A* **103**, 043316 (2021).

[38] Astrakharchik, G. E. & Malomed, B. A. Dynamics of one-dimensional quantum droplets. *Phys. Rev. A* **98**, 013631 (2018).

[39] Mistakidis, S. I., Mithun, T., Kevrekidis, P. G., Sadeghpour, H. R. & Schmelcher, P. Formation and quench of homonuclear and heteronuclear quantum droplets in one dimension. *Phys. Rev. Res.* **3**, 043128 (2021).

[40] Tononi, A., Wang, Y. & Salasnich, L. Quantum solitons in spin-orbit-coupled Bose–Bose mixtures. *Phys. Rev. A* **99**, 063618 (2019).

[41] Cheiney, P. et al. Bright soliton to quantum droplet transition in a mixture of Bose–Einstein condensates. *Phys. Rev. Lett.* **120**, 135301 (2018). 29694210 10.1103/PhysRevLett.120.135301

[42] Pathak, M. R. & Nath, A. Dynamics of quantum droplets in an external harmonic confinement. *Sci. Rep.* **12**, 6904 (2022). 35484174 10.1038/s41598-022-10468-6 PMC9050709

[43] Malomed, B. A. The family of quantum droplets keeps expanding. *Front. Phys.* **16**, 22504 (2021).

[44] Khan, A. & Debnath, A. Quantum droplet in lower dimensions. *Front. Phys.* **10**, 887338 (2022).

[45] Gangwar, S., Ravisankar, R., Muruganandam, P. & Mishra, P. K. Dynamics of quantum solitons in Lee–Huang–Yang spin-orbit-coupled Bose–Einstein condensates. *Phys. Rev. A* **106**, 063315 (2022).

[46] Gangwar, S., Ravisankar, R., Mistakidis, S. I., Muruganandam, P. & Mishra, P. K. Spectrum and quench-induced dynamics of spin-orbit-coupled quantum droplets. *Phys. Rev. A* **109**, 013321 (2024).

[47] Mithun, T., Maluckov, A., Kasamatsu, K., Malomed, B. A. & Khare, A. Modulational instability, inter-component asymmetry, and formation of quantum droplets in one-dimensional binary Bose gases. *Symmetry* **12**, 174 (2020).

[48] Debnath, A. & Khan, A. Investigation of quantum droplets: An analytical approach. *Ann. Phys.* **533**, 2000549 (2021).

[49] Hu, H. & Liu, X. J. Microscopic derivation of the extended Gross–Pitaevskii equation for quantum droplets in binary Bose mixtures. *Phys. Rev. A* **102**, 043302 (2020).

[50] Zin, P., Pylak, M. & Gajda, M. Zero-energy modes of two component Bose–Bose droplets. *New J. Phys.* **23**, 033022 (2021).

[51] Cui, X. L. Spin-orbit-coupling-induced quantum droplet in ultracold Bose-Fermi mixtures. *Phys. Rev. A* **98**, 023630 (2018).

[52] Li, G. et al. Two-dimensional anisotropic vortex quantum droplets in dipolar Bose–Einstein condensates. *Front. Phys.* **19**, 22202 (2024).

[53] Kartashov, Y. V., Malomed, B. A., Tarruell, L. & Torner, L. Three-dimensional droplets of swirling superfluids. *Phys. Rev. A* **98**, 013612 (2018).

[54] Debnath, A., Khan, A. & Malomed, B. Interaction of one-dimensional quantum droplets with potential wells and barriers. *Commun. Nonlinear Sci. Numer. Simul.* **126**, 107457 (2023).

[55] Bhatia, S., Kumar, C. N. & Nath, A. Investigation of one-dimensional quantum droplets in a temporally perturbed external harmonic trap. *Phys. Lett. A* **492**, 129228 (2023).

[56] Das, S. & Nath, A. Quantum droplet speed management and supersolid behavior in external harmonic confinement. http://arxiv.org/abs/2407.10463.

[57] Morera, I., Astrakharchik, G. E., Polls, A. & Juliá-Díaz, B. Universal dimerized quantum droplets in a one-dimensional lattice. *Phys. Rev. Lett.* **126**, 023001 (2021). 33512190 10.1103/PhysRevLett.126.023001

[58] Morera, I., Astrakharchik, G. E. & Polls, A. Quantum droplets of bosonic mixtures in a one-dimensional optical lattice. *Phys. Rev. Res.* **2**, 022008(R) (2020).

[59] Pathak, M. R. & Nath, A. Formation of matter-wave droplet lattices in multi-color periodic confinements. *Symmetry* **14**, 963 (2022).

[60] Pathak, M. R. & Nath, A. Droplet to soliton crossover at negative temperature in presence of biperiodic optical lattices. *Sci. Rep.* **12**, 6904 (2022). 36309597 10.1038/s41598-022-23026-x PMC9617923

[61] Zhou, Z., Yu, X., Zou, Y. & Zhong, H. H. Dynamics of quantum droplets in a one-dimensional optical lattice. *Commun. Nonlinear Sci. Numer. Simul.* **78**, 104881 (2019).

[62] Nie, Y., Zheng, J.-H. & Yang, T. Spectra and dynamics of quantum droplets in an optical lattice. *Phys. Rev. A* **108**, 053310 (2023).

[63] Zhao, F. Y. et al. Discrete quantum droplets in one-dimensional optical lattices. *Chaos Solitons Fractals* **152**, 111313 (2021).

[64] Dong, L. W. et al. Multi-stable quantum droplets in optical lattices. *Nonlinear Dyn.* **102**, 303 (2020).

[65] Zheng, Y.-Y. et al. Quantum droplets in two-dimensional optical lattices. *Front. Phys.* **16**, 22501 (2021).

[66] Zhang, X. L. et al. Semidiscrete quantum droplets and vortices. *Phys. Rev. Lett.* **123**, 133901 (2019). 31697515 10.1103/PhysRevLett.123.133901

[67] Semeghini, G. et al. Self-bound quantum droplets of atomic mixtures in free space. *Phys. Rev. Lett.* **120**, 235301 (2018). 29932719 10.1103/PhysRevLett.120.235301

[68] Burt, E. A. et al. Coherence, correlations, and collisions: What one learns about Bose–Einstein condensates from their decay. *Phys. Rev. Lett.* **79**, 337 (1997).

[69] Filho, V. S., Holz, S. M. & Tomio, L. Dynamics of Bose–Einstein condensates with atomic pumping and dissipative processes. *Phys. Lett. A* **372**, 6778 (2008).

[70] Mewes, M.-O. et al. Collective excitations of a Bose–Einstein condensate in a magnetic trap. *Phys. Rev. Lett.* **77**, 988 (1996). 10062962 10.1103/PhysRevLett.77.988

[71] Lahaye, T. et al. d-Wave collapse and explosion of a dipolar Bose–Einstein condensate. *Phys. Rev. Lett.* **101**, 080401 (2008). 18764592 10.1103/PhysRevLett.101.080401

[72] Kagan, Y., Muryshev, A. E. & Shlyapnikov, G. V. Collapse and Bose–Einstein condensation in a trapped Bose gas with negative scattering length. *Phys. Rev. Lett.* **81**, 933 (1998).

[73] Kundu, N., Nath, A., Bera, J., Ghosh, S. & Roy, U. Synergy between the negative absolute temperature and the external trap for a Bose–Einstein condensate under optical lattices. *Phys. Lett. A* **427**, 127922 (2022).

[74] Windpassinger, P. & Sengstock, K. Engineering novel optical lattices. *Rep. Prog. Phys.* **76**, 086401 (2013). 23828639 10.1088/0034-4885/76/8/086401

[75] Katsimiga, G. C. et al. Interactions and dynamics of one-dimensional droplets, bubbles and kinks. *Condensed Matter* **8**, 67 (2023).

[76] Katsimiga, G. et al. Solitary waves in a quantum droplet-bearing system. *Phys. Rev. A* **107**, 063308 (2023).

[77] Edmonds, M. Dark quantum droplets and solitary waves in beyond-mean-field Bose–Einstein condensate mixtures. *Phys. Rev. Res.* **5**, 023175 (2023).

[78] Du, X., Fei, Y., Chen, X.-L. & Zhang, Y. Ground-state properties and Bogoliubov modes of a harmonically trapped one-dimensional quantum droplet. *Phys. Rev. A* **108**, 033312 (2023).

[79] Kengne, E., Liu, W.-M. & Malomed, B. A. Spatiotemporal engineering of matter-wave solitons in Bose–Einstein condensates. *Phys. Rep.* **899**, 1–62 (2021).

